# Six Hydrophobins Are Involved in Hydrophobin Rodlet Formation in *Aspergillus nidulans* and Contribute to Hydrophobicity of the Spore Surface

**DOI:** 10.1371/journal.pone.0094546

**Published:** 2014-04-10

**Authors:** André Grünbacher, Tanja Throm, Constanze Seidel, Beatrice Gutt, Julian Röhrig, Timo Strunk, Paul Vincze, Stefan Walheim, Thomas Schimmel, Wolfgang Wenzel, Reinhard Fischer

**Affiliations:** 1 Karlsruhe Institute of Technology (KIT) - Campus South, Institute for Applied Biosciences (IAB), Department of Microbiology, Karlsruhe, Germany; 2 KIT - Campus North, Institute of Nanotechnolgy (INT), Eggenstein-Leopoldshafen, Germany; 3 KIT - Campus North, Institute of Applied Physics and INT, Eggenstein-Leopoldshafen, Germany; Woosuk University, Republic Of Korea

## Abstract

Hydrophobins are amphiphilic proteins able to self-assemble at water-air interphases and are only found in filamentous fungi. In *Aspergillus nidulans* two hydrophobins, RodA and DewA, have been characterized, which both localize on the conidiospore surface and contribute to its hydrophobicity. RodA is the constituent protein of very regularly arranged rodlets, 10 nm in diameter. Here we analyzed four more hydrophobins, DewB-E, in *A. nidulans* and found that all six hydrophobins contribute to the hydrophobic surface of the conidiospores but only deletion of *rodA* caused loss of the rodlet structure. Analysis of the rodlets in the *dewB-E* deletion strains with atomic force microscopy revealed that the rodlets appeared less robust. Expression of DewA and DewB driven from the *rodA* promoter and secreted with the RodA secretion signal in a strain lacking RodA, restored partly the hydrophobicity. DewA and B were able to form rodlets to some extent but never reached the rodlet structure of RodA. The rodlet-lacking *rodA*-deletion strain opens the possibility to systematically study rodlet formation of other natural or synthetic hydrophobins.

## Introduction

Hydrophobins are small, filamentous fungal proteins, characterized by their distinctive pattern of cysteine residues that form four intramolecular disulfide bridges. Besides the conserved cysteines, overall sequence similarity between different hydrophobins is generally very low. Two classes of hydrophobins have been defined according to sequence characteristics, which differ in hydropathy properties [1]. Proteins of either class are able to form stable membranes. However, class I hydrophobins form membranes, which can only be dissolved in organic solvents and 2% SDS, whereas class II hydrophobins can be easily dissolved in aqueous ethanol (60%) or 2% SDS [2]. Normally fungi contain either hydrophobins of class I or of class II, although it was reported that four of the six hydrophobins of *Cladosporium fulvum* are class I and two are class II hydrophobins [3]. Likewise, not all six hydrophobins of *A. nidulans* may be classified unambiguously to one of the two classes [4].

The name *hydrophobin* was given by Wessels and colleagues, who examined genes that are expressed during fruiting body formation in *Schizophyllum commune* [5]. After the discovery in *S. commune,* hydrophobins have been identified in several other fungi, among which was *Aspergillus nidulans* [6]. Here, hydrophobin was discovered in a screen for differentially expressed genes during conidiospore development. The first one being characterized was *rodA*. The protein localized to the surface of conidiospores. Electron microscopy images revealed a most interesting and striking property of this hydrophobin, namely the formation of highly ordered rodlets on the spore surface [6]. Later, a second hydrophobin was discovered and named *dewA*. The absence of DewA did not lead to a loss of the rodlet structure on the spore surface, suggesting that RodA is the main constituent. Nevertheless, DewA appeared to contribute to the hydrophobicity of the spore surface [7]. In *A. fumigatus* two hydrophobins, RodA and RodB, were characterized. Whereas RodA appeared to be the major rodlet-forming hydrophobin on the spore, RodB plays a distinct role in the structure of the conidial cell wall [8], [9]. Later studies showed that the hydrophobic rodlet layer on the spore surface helps the fungus to hide from the immune system in patients [10]. In *Beauveria bassiana* also two hydrophobins were found on the spore surface, but only hydrophobin 1 (Hyd1) is able to form rodlets. However, in the absence of the second hydrophobin, Hyd1 rodlets were truncated and incomplete, suggesting interaction or dependence of the two hydrophobins [11].

Hydrophobins (e.g. SC3 of *S. commune*) do not only form monolayers *in vivo* but also have the property to assemble at a gas-water interface in SDS (sodium dodecyl sulfate) into an insoluble amphipathic film. It was suggested, that the amphiphilicity is the driving force for the self-assembly of hydrophobins [12]. Electron microscopic analyses of coated artificial surfaces showed that these films consist of ordered microfibrils similar to the rodlet structures on the surface of many molds [13], [14].

Already soon after their discovery scientists recognized the high technological potential of these amphiphilic proteins [2]. In 2006 the *N. crassa* class I hydrophobin EAS was purified after expression in *E. coli* and in 2009 the BASF SE company succeeded to express *A. nidulans* DewA in the bacterial system [15], [16]. Along with the possibility to produce high amounts of hydrophobin, several applications were feasible. In addition to the application of natural hydrophobin, the proteins can be modified to functionalize surfaces. For instance, fusion of a RGD sequence or the laminin globular domain LG3 binding motif to the N-terminus of DewA stimulated adhesion of mesenchymal stem cells [17].

In order to improve the application, an important key could be the understanding of the function of several hydrophobins in one organism. In terms of technical applications, but also from a basic knowledge point of view, it would be highly desirable to understand rodlet formation of this class of proteins. Here, we tested six hydrophobins of *A. nidulans* for their capability to form rodlets *in vivo* and found that all six were able to self-assemble rodlets to some extent but that only RodA formed stable and highly ordered rodlets.

## Results

### Six hydrophobins in *A. nidulans*

**Figure 1:**
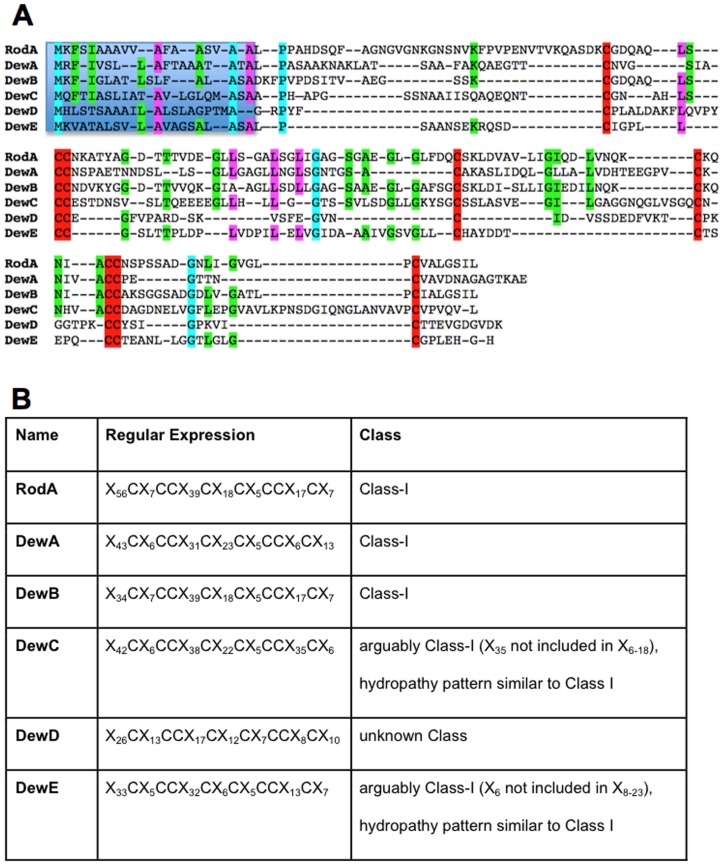
Alignment and structure of the six *A. nidulans* hydrophobins. (**A**) Alignment of RodA with the other five hydrophobins. Sequences were aligned manually. The blue box shows the N-terminal signal peptide, 8 characteristic cysteines of each hydrophobin (red), which appear in the characteristic pattern 1-2-1-1-2-1. The different colors represent the homology between the hydrophobin open reading frames. Blue indicates 100% identity, pink 71% and green 57% homology. Besides the conserved cysteine residues, overall sequence similarity of the hydrophobins is very low. (**B**) Assignment of hydrophobin proteins to hydrophobin classes. The analyzed hydrophobins all feature the common eight cysteine-motif and are members of the Class-I hydrophobin family with the exception of DewD.

The *A. nidulans* genome encodes six hydrophobins, two of which (RodA and DewA) were already characterized [6], [7]. The four new proteins (AspGD AN1837, 6401, 0940, 7539) were named DewB, C, D and E, respectively. They are between 101 and 162 amino acids in length and share a signal peptide at their N-termini. The intron-exon borders were confirmed by comparison of the genomic DNA sequence with RNAseq data. RodA, DewA and DewB were classified as class I hydrophobins, whereas the remaining three as *intermediate*. In RodA and DewB a GPI anchor was predicted using the bigPI Predictor and the fungal prediction algorithm [23]. The other four hydrophobins did not have this motif. In order to visualize the differences between the different hydrophobins, we aligned them all but also pairwise with RodA. Due to the minimal sequence conservation conventional multiple sequence alignment tools, such as Clustal Omega [24], T-Coffee [25] or Muscle [26] failed to align the characteristic cysteine residues (**Fig. 1**). Therefore, the AlignMe server [27] was used to generate sequence alignments based on similar hydrophobicity patterns. Alignments were generated pairwise towards RodA with a gap-opening penalty of 10 and a gap extension penalty of 5. The BLOSUM62 matrix [28] was used with a weight of 10 as a sequence similarity score in conjunction to a hydrophobicity similarity score using the Kyte-Doolittle scale [29] with a window size of 7 and a weight of 15.

**Figure 2:**
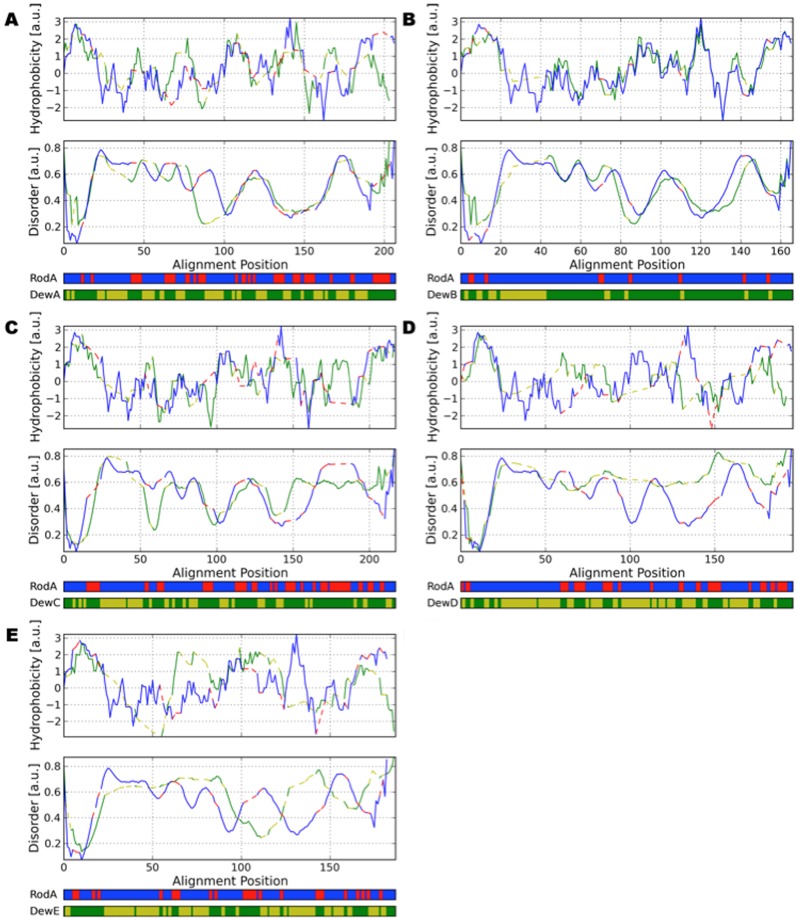
Pairwise alignments for all hydrophobin sequences in comparison to RodA. (**A**) Up: Hydrophobicity of the alignment of RodA with DewA. In comparison with DewB, DewA does not share as much similarity in the hydrophobicity pattern with RodA. The two hydrophobic unstructured loops are conserved; DewA features a stronger hydrophobic region towards the N-Terminus. Middle: Predicted disorder for RodA/DewA. Similar to RodA, intrinsically disordered regions are predicted for two large loops towards the C-Termini. (**B**) Up: Hydrophobicity of the alignment of RodA with DewB. Due to their similarity in sequence RodA and DewB share similar hydropathicity patterns. A large gap is observed in the sequence of DewB towards the N-Terminus. Middle: Predicted disorder for RodA/DewB. Both proteins share a very similar predicted disorder pattern. The RodA sequence aligned to the gap in DewB is predicted to be largely unstructured. The two unstructured loops between the cysteines towards the C-Terminus can be clearly discerned. (**C**) Up: Hydrophobicity of the alignment of RodA with DewC. Especially towards the C-Terminus DewC exhibits a hydrophobicity pattern unlike the one from RodA. Middle: Predicted disorder for RodA/DewC. A large unordered region was predicted for the DewC sequence. Like all other analyzed hydrophobins except for DewD, the large unstructured loop (alignment positions 100-150) is conserved. (**D**) Up: Hydrophobicity of the alignment of RodA with DewD. DewD exhibits a hydrophobicity pattern unlike all the other hydrophobins. Especially the characteristic loop between alignment positions 125 and 150 is missing. Middle: Predicted disorder for RodA/DewD. DewD is predicted to exhibit a very high amount of intrinsic disorder. (**E**) Up: Hydrophobicity of the alignment of RodA with DewE. The hydrophobicity pattern of DewE is unlike that of RodA with a second hydrophobic region in the middle of the sequence. Middle: Predicted disorder for RodA/DewE. The two short unordered loops of DewE are shifted in comparison with RodA. For all images: Down: Sequence Alignment Blue/Red RodA/Gaps, Green/Yellow Other/Gaps

Intrinsic disorder was predicted for each sequence using the DisEMBL disorder prediction [30] with a smoothing frame of 8 residues, a minimum peak width of 8 residues and a maximum join distance of 4 residues. The pairwise alignments to RodA show that all studied hydrophobin sequences share the eight-cysteine motif characteristic for Class-I hydrophobins [31], but larger sequence similarity to RodA is only observed for DewB (**Fig. 2**). Consensus in the sequences occurs especially for hydrophobic amino acids.

Littlejohn et al. reported even about four more putative hydrophobins [32]. However, these proteins were in general longer than the so-far identified hydrophobins and three of them contain a predicted GPI anchor. In our view it is not yet clear, whether these could be hydrophobins or not. Therefore, we have not analyzed them in this study.

### Expression of the hydrophobins

**Figure 3:**
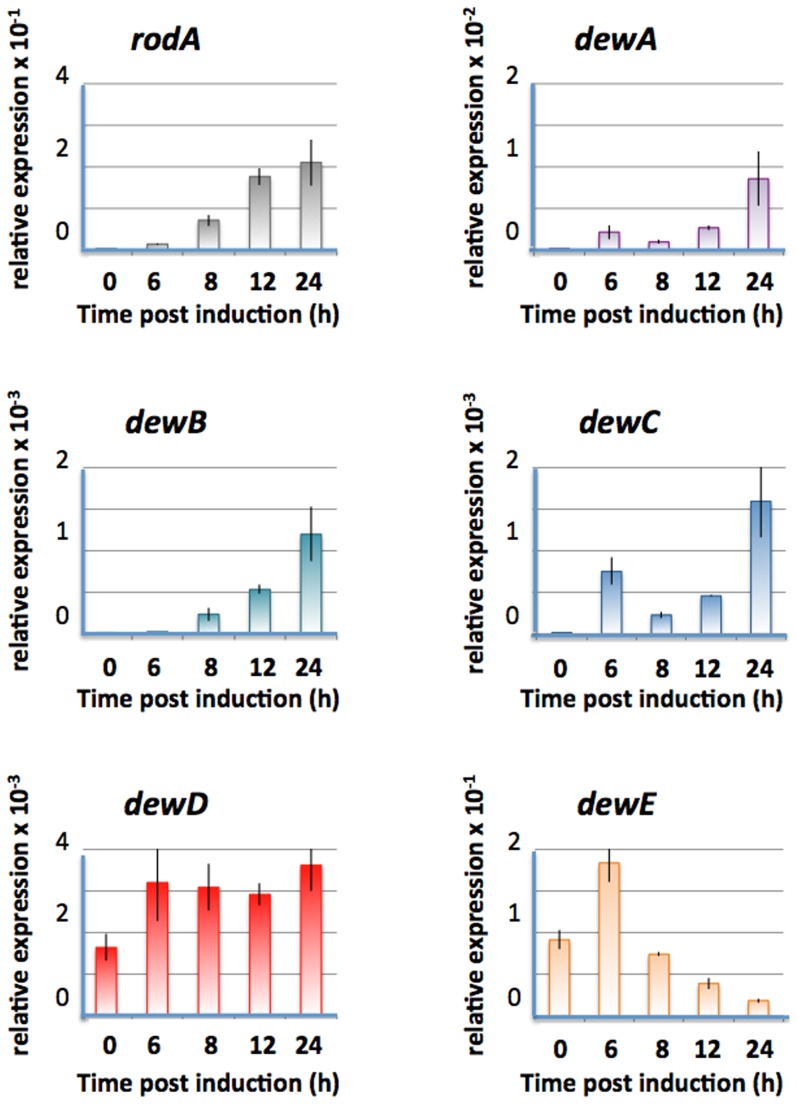
Expression patterns of *A. nidulans* hydrophobins (rodA, dewA-E) during development. TN02A3 was grown in liquid medium over night before shifting to solid minimal medium. RNA was isolated and the mRNA of the corresponding genes quantified by real time PCR. The expression was normalized to histone H2b. Error bars represent the standard error (SEM).

In order to study the function of the newly identified putative hydrophobins DewB-E, the expression of the genes was studied in vegetative hyphae and during asexual development. Mycelium of strain TN02A3 was grown in liquid medium (supplemented with uracil, uridine and pyrodoxin) for 12 h at 37°C. During this time the strain acquired developmental competence. In order to synchronize asexual development, the competent mycelium was filtered and exposed to an agar surface. After defined time points, mycelium was harvested and processed for Northern blot and/or real time RT PCR analyses. For comparison, *dewA* and *rodA* were monitored. All hydrophobins were expressed after 12 to 24 hours post induction of asexual development, which correlates with the development of metulae and phialides (**Fig. 3**). However, whereas *rodA, dewA, dewB*, and *dewC* were not expressed in hyphae and strongly induced during development, *dewD* and *dewE* were already expressed in vegetative hyphae (time point 0). In the case of *dewE* the expression decreased after a peak at 6 h post induction.

**Figure 4:**
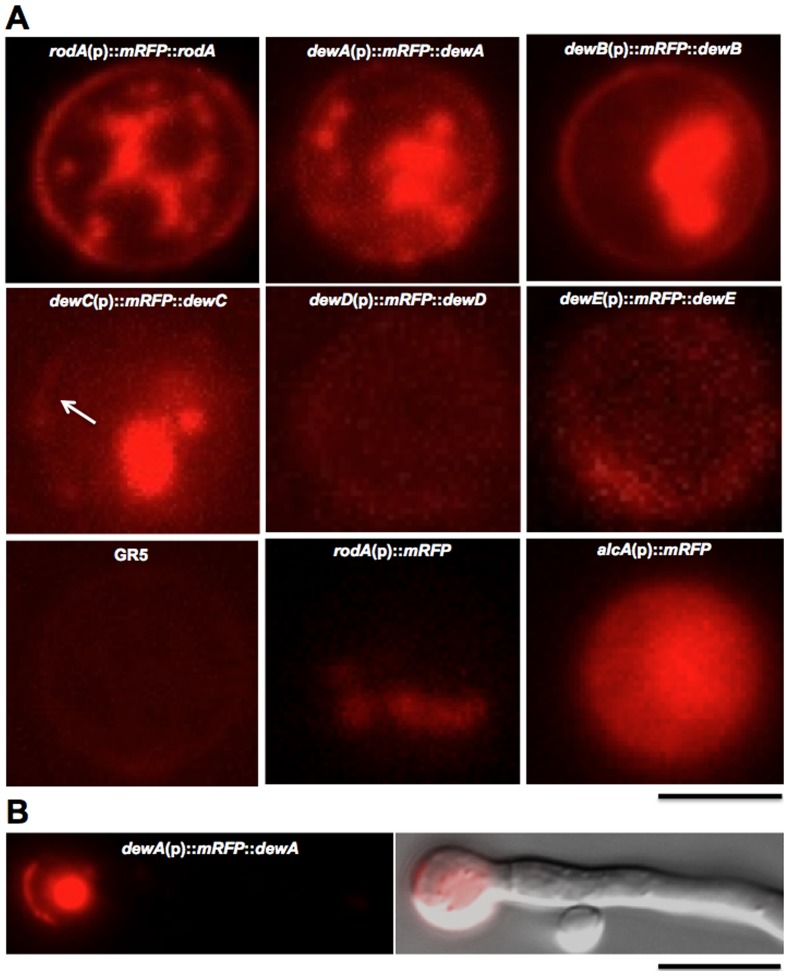
Localization of mRFP-tagged hydrophobins. Constructs (as indicated) were transformed into GR5 or TN02A3. Strains: SCOS170-SCOS175, SAGR19a, and STT08. Scale bar, 2 μm in **A** and 5 μm in **B**.

Next, the corresponding proteins were localized as fusion proteins with red fluorescent protein (mRFP). All genes were N-terminally tagged and expressed from their own promoter (pAGR13-18). The endogenous signal peptide at the N-terminus has been preserved. The constructs were transformed into GR5 (*dewA-C, E*) or TN02A3 (*rodA*) or RMS019 (*dewD*). The copy number was not determined. For all hydrophobins a fluorescent signal was detected at the spore surface, although the signal intensity at the spore surface varied and typical pictures were selected for the figure (**Fig. 4** **, A**). In the case of DewC, D, and E, the signal was very weak at the spore surface and is hardly visible in the pictures. In order to distinguish between auto-fluorescence and a true mRFP signal, several controls were included into the analysis. The recipient strains did not show any fluorescence. When the *rodA* promoter with the signal peptide was fused to mRFP, only very weak fluorescence was detected in the cytoplasm, suggesting that the secretion signal of RodA worked. In the absence of the secretion signal and expression under the control of the *alcA* promoter, strong fluorescence was detected in the cytoplasm (**Fig. 4** **, B**).

Germlings showed only fluorescence at the spore surface and not along the hyphae (**Fig. 4** **, C**). Older hyphae and conidiophores displayed strong auto-fluorescence (data not shown). Therefore, protein expression and localization could not be determined in these structures. Especially for DewD and DewE it would have been very desirable to monitor the localization in vegetative hyphae.

### All six hydrophobins contribute to the hydrophobicity of the spore surface

To unravel the molecular function of *dewB-E*, corresponding deletion strains were constructed. Using a fusion-PCR-based approach, we replaced the open reading frames in the hydrophobin wild type strain, TN02A3, by the nutritional marker *pyrG* of *Aspergillus fumigatus.* The deletion event was confirmed by PCR and Southern blotting (data not shown). Each deletion strain was re-complemented with a linear PCR fragment of the hydrophobin open reading frames including 1 kb up- and downstream sequence. The PCR products were co-transformed with a selection marker plasmid (pNZ11, pCK17). Because the recipient strain contained the nkuA mutation, homologous integration of the fragment can be assumed. Indeed, the strains were uracil auxotrophic after transformation.

**Figure 5:**
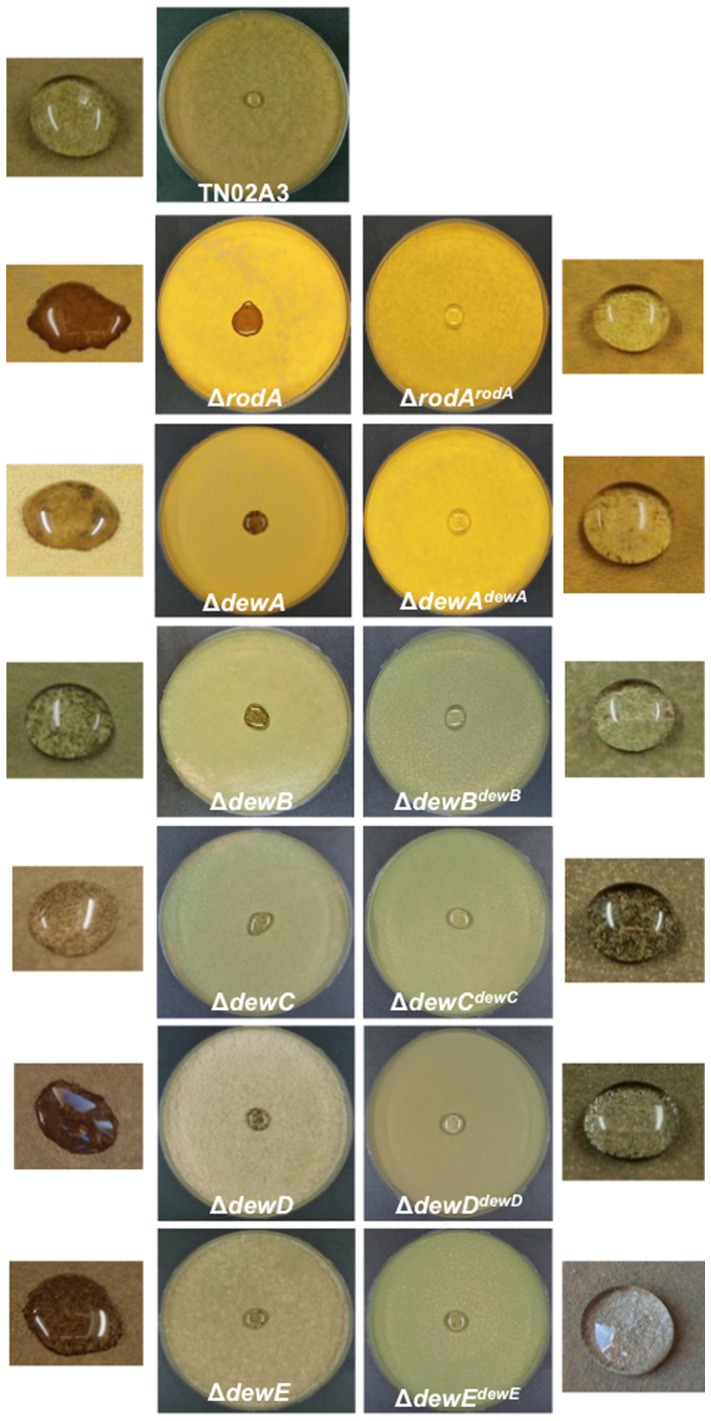
Hydrophobicity tests of wild type, all hydrophobin deletion strains (left) and their corresponding re-complementations (right). Strains were inoculated as spore suspensions (10^6^ per plate) using glass beads to obtain very homogenous lawns and grown for 40 h at 37°C. The bigger pictures show the aspect of the entire agar plate covered with a lawn of the corresponding strain and with a droplet in the middle of the plate. The small pictures show enlarged droplets. 10 μl of a detergent solution (0.2% SDS, 50 mM EDTA) were dropped onto the surface of a colony. Lower hydrophobicity is indicated by soaking of the liquid into the colony. Besides change of the contact angle, the effect is easily visible through the brown color. This is due to the color of conidiophore stalks and vesicles. If the droplet does not soak into the colony, the brown color is hidden by the yellow spore color. Strains: TN02A3, RMS019, TMS027, STT01, STT02, SAGR01, SAGR12, SAGR02, 03, 04, 05, 11, 13.

**Figure 6:**
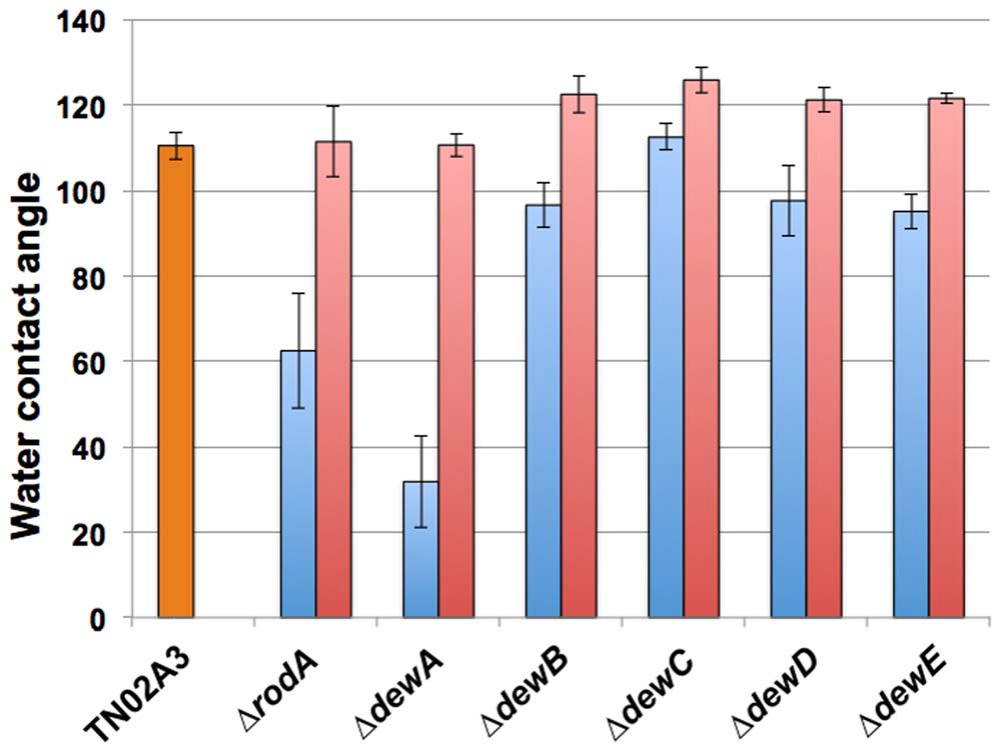
Water contact angle measurement of all hydrophobin deletion strains and their corresponding re-complementations in comparison to wild type. 150 μl of a detergent solution (0.2% SDS, 50 mM EDTA) were dropped on the surface of a lawn. The angle (degree) between the surface and the drop was measured. The mean of 10 measurements is displayed. The error bar represents the standard deviation.

Because both, RodA and DewA contribute to the hydrophobicity of the spore surface, the newly created deletion strains and corresponding re-complemented strains were compared to wild type with regards to their hydrophobicity (**Fig. 5**). To this end, a drop of 150 μl detergent (0.2% SDS/50 mM EDTA solution) was spotted onto the surface of a lawn on an agar plate. Detergent was added to lower the surface tension. In case the spores are more hydrophilic, the droplet is soaked into the colony and the brownish color of the conidiophore stalk and vesicle becomes visible. In all cases colonies appeared more hydrophilic, although in the case of DewC the effect was only very little. All re-complemented strains were again hydrophobic (**Fig. 5**). In order to get a more quantitative value, contact angles of the corresponding droplets were measured. This essentially confirmed the visual inspection (**Fig. 6**). In the case of DewC, only a small difference in comparison to wild type was observed. Surprisingly, the contact angle of the colonies lacking DewA was even lower than the contact angle of RodA. In the re-complemented strains the angle was sometimes slightly higher than in wild type.

**Figure 7:**
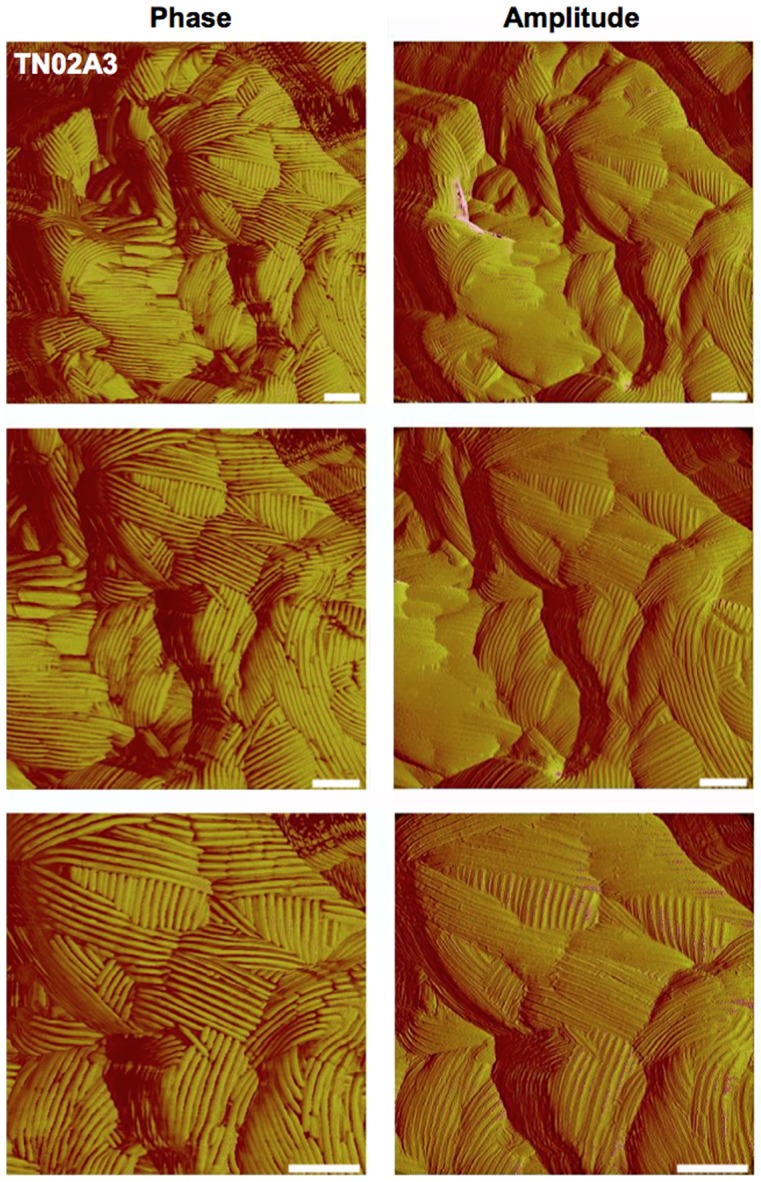
Phase and amplitude images of the spore surface of the wild type TN02A3 taken with atomic force microscopy (AFM) in tapping mode. The ordered rodlet structure is clearly visible. For TN02A3 phase as well as amplitude images show very good quality. Scale bars, 100

**Figure 8:**
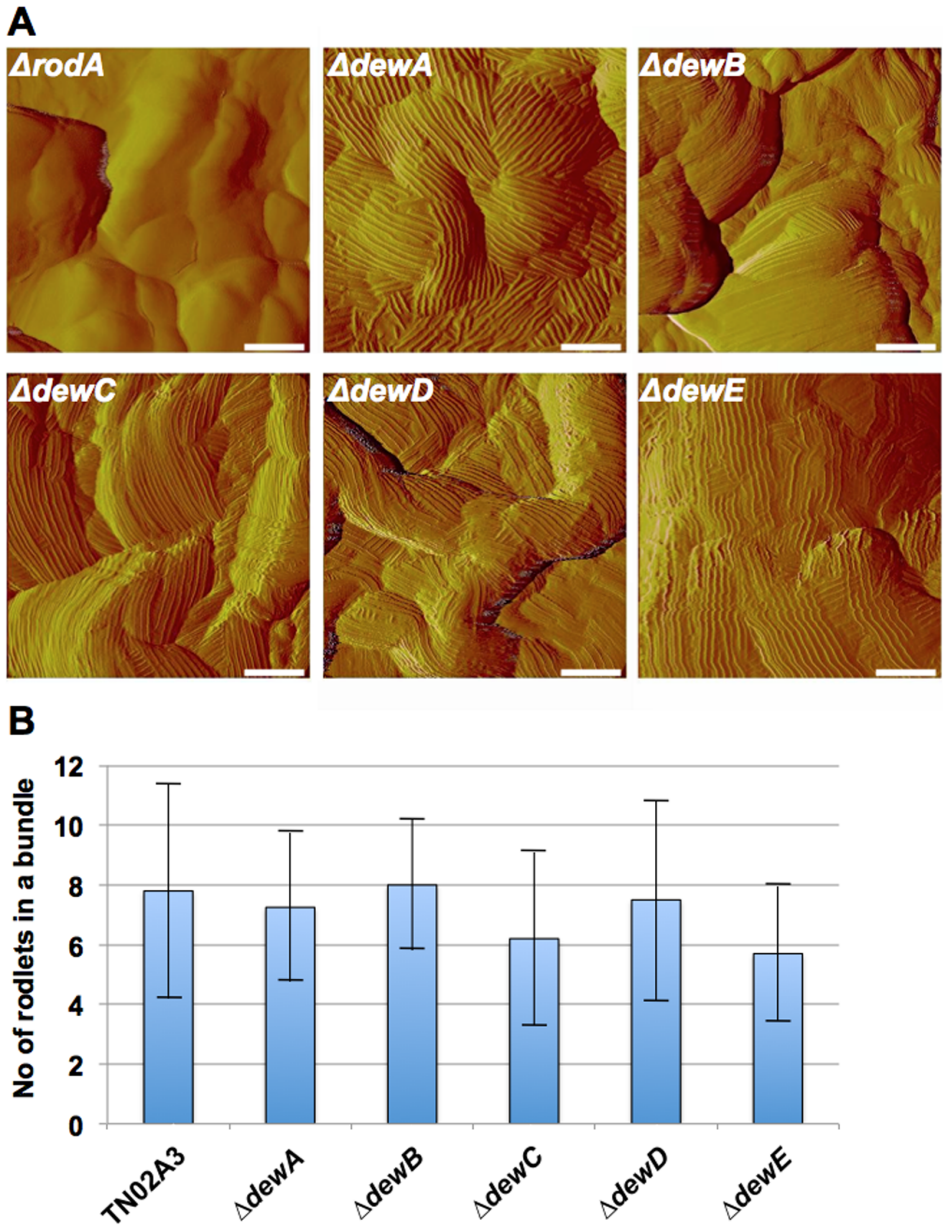
Characterization of all hydrophobin deletion strains. (**A**) AFM amplitude images of the spore surfaces. Whereas the *rodA* deletion strain (RMS019) does not show any rodlets, the dewA-E deletion strains (TMS027, STT01, STT02, SAGR01, SAGR12) display rodlets. Scale bars, 100 nm. (**B**) Determination of the number of rodlets per bundle. For each strain 20 bundles were analyzed and the mean value displayed. The error bars represent the standard deviation. Strains see Figure legend of Fig. 5.

Next we analyzed the spore surface using atomic force microscopy (AFM). Spores were immobilized on polished silicon wavers and rodlets visualized in the phase and the amplitude modus. The rodlet structure appeared very robust and the quality of the images was always very good (**Fig. 7**). When the spore surface of all mutants was analyzed, we found that only the *rodA*-deletion strain did not show any rodlets. The *dewA-E*-deletion strains clearly displayed rodlets (**Fig. 8**). However, the rodlets appeared less robust and nice images could only be obtained in the amplitude modus. This suggests that DewA-E play roles in RodA rodlet formation or the organization or stability of the rodlets.

### Analysis of rodlet formation on the spore surface

**Figure 9:**
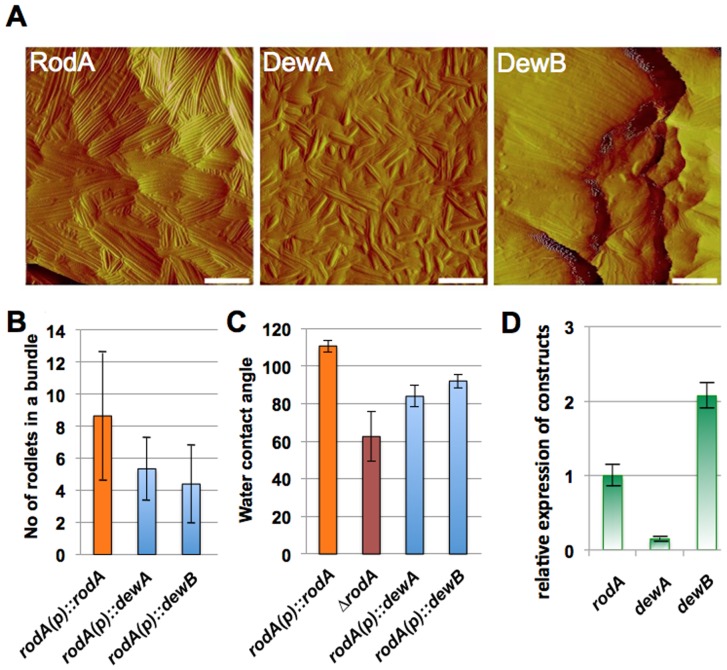
(**A**) Analysis of the capability of hydrophobins DewA and DewB to form rodlets on the spore surface of a RodA lacking strain. The *ΔrodA* strain was used as expression platform. DewA and B were expressed from the *rodA* promoter and secreted with the signal peptide of RodA. As a control *rodA* was expressed in the same way. Strains: SAGR14, SAGR06, and 07. Scale bars, 100 nm. (**B**) Comparison of the number of rodlets per bundle. The number of rodlets was counted in 20 bundles. The mean value is displayed and the standard deviation indicated. (**C**) Water contact angle measurement of the *rodA-*expressing strain (SAGR14), the *rodA*-deletion strain and the Δ*rodA* strain transformed with the other two hydrophobins. The mean of ten independent measurements is displayed with the standard deviation. (**D**) Expression of *rodA, dewA* and *dewB* in the corresponding strains quantified by real time RT PCR. Strains were grown on the surface of liquid minimal medium for 24 h. Expression was normalized to histone 2B minus the normalized expression of the two hydrophobins in a *rodA*-deletion strain (RMS019). RodA was then set as one. In this way the value reflects the expression due to the activity of the *rodA* promoter. The mean of two technical and three biological replicates is shown with the standard error.

The most intriguing property of RodA is its rodlet formation. Because deletion of any of the other hydrophobins did change the rodlet layer only slightly, it remained open whether DewA-E are able to form rodlets on the spore surface or not. The fact that in the absence of RodA no rodlet structures were visible, suggests that DewA-E do not form rodlets in this strain. This could be due to the lower expression level in comparison to *rodA* (**Fig. 3**). In order to change the expression level, we placed DewA and DewB under the control of the *rodA* promoter (including the signal peptide) und transformed it into the Δ*rodA* strain. As a control the *rodA* open reading frame was cloned in the same way. AFM analyses of the spore surfaces revealed that DewA and B were able to form rodlets to some extent (**Fig. 9** **, A**). However, the surface looked clearly different from the control strain (RodA) and the bundles contained less rodlets (**Fig. 9** **, B**). Water contact angle measurements revealed a slight increase of the hydrophobicity in the transformed strains. The expression of the constructs was analyzed by real time PCR. Whereas the expression level for *dewA* was lower than for *rodA*, it was higher in the case of *dewB*.

## Discussion

Hydrophobins are a fascinating class of fungal proteins and their role(s) are not fully understood. Normally each fungus contains a number of different hydrophobins [12]. *Cladosporium fulvum* (responsible for the velvet and brown spot disease) harbors at least six hydrophobins. HCf-1-4 are typical class I hydrophobins and HCf-5 and HCf-6 belong to class II hydrophobins [3]. The presence of several hydrophobin genes in one organism has two possible explanations. Either they are expressed during different stages of development or in response to changing environmental conditions and thus fulfill distinct functions. This appears to be the case in *C. fulvum*, where HCf-6 is required for adhesion [33]. Alternatively, they can largely be able to complement each other [12]. Even if they are important for different morphogenetic processes, they may be able to substitute for each other. One example is the phytopathogenic fungus *Magnaporthe grisea*. Hydrophobins, such as *S. commune* SC1 and SC4, or *A. nidulans* RodA and DewA or EAS of *N. crassa* expressed from the *mpg1*-promoter were all able to at least partially complement the *mpg-1* mutation [34]. Here, we compared the six different hydrophobins of *A. nidulans*. They were all expressed during asexual development and localized to the spore surface. DewD and DewE were also expressed in hyphae. They all contributed to colony hydrophobicity, which can be due to a reduced hydrophobicity of the spores or of the hyphae. Only the absence of RodA caused the loss of the conidial rodlet structure. Nevertheless, we noticed some minor effects on the integrity of the RodA rodlet layer if one of the other hydrophobins was missing. These results suggest that DewA-E are required for proper rodlet formation of RodA. A similar effect has been described recently in *B. bassiana* [11]. The structural basis currently remains elusive. One possibility could be a direct physical and/or functional interaction between the different hydrophobins on the spore surface. In order to test this, bi-molecular complementation tests were performed. However, they were all negative (data not shown). This could mean that they do not interact or that the method does not work outside the cell. On the other hand it was reported in *A. fumigatus* that the cell wall structure changes upon deletion of *rodB* [8]. Likewise, DewA-E could be involved in cell wall formation and thus preparation of RodA assembly on the surface.

One other interesting aspect of this study is that RodA could not be substituted through DewA or DewB. Although transformation of either of these hydrophobins into a *ΔrodA* strain increased the hydrophobicity, and both were able to form some rodlet-like structures, none was able to really form rodlets similar to the ones formed by RodA. This is even more surprising given that DewA is able to form stable rodlets *in vitro* [13]. One difference between DewA and RodA is the presence of a GPI anchor in RodA. This covalently links the hydrophobin to the cell wall. However, a GPI anchor was found in DewB, which was also unable to form extensive rodlets on the spore surface. One strategy to solve the interesting question of rodlet formation could be the construction of chimeric proteins between DewA and RodA, which could be easily tested for rodlet formation using the *ΔrodA* strain as recipient.

## Materials and Methods

### Strains, plasmids and culture conditions

Supplemented minimal (MM) and complete media (CM) for *A. nidulans* were prepared as described, and standard strain construction procedures are described by Hill and Käfer [18]. A list of *A. nidulans* strains used in this study is given in **Table S1**. Standard laboratory *Escherichia coli* strains (XL-1 blue, Top 10 F') were used. Plasmids are listed in **Table S2** and oligonucleotides in **Table S3**.

### Molecular techniques

Standard DNA transformation procedures were used for *A. nidulans* [19] and *E. coli* [20]. For PCR experiments, standard protocols were applied using a Biometra Personal cycler (Biometra, Göttingen) for the reaction cycles. DNA sequencing was done commercially (Eurofins-MWG-operon, Ebersberg, Germany). Genomic DNA was extracted from *A. nidulans* with the DNeasy Plant Mini Kit (Qiagen, Hilden, Germany). DNA analyses (Southern hybridizations) were performed as described by [20]. Western blot was performed with the MiniProtean system (Bio-Rad) following the manufacturers instructions.

Gene deletions were done with PCR fragments of fusion PCR constructs as described [21]. Deletion cassettes were obtained from the Fungal Genetics stock center for *dewB, D,* and *E.* The cassette for *dewC* was generated by fusion PCR with *Sfi*I restriction sites and *A. fumigatus pyrG* as selection marker [22]. *A. nidulans* TN02A3 was used as the recipient strain for all plasmids. Homologous integration and gene replacements were confirmed by Southern blot and PCR (data not shown). All deletion strains were re-complemented by the *open reading frame* of the corresponding hydrophobin. Re-complementation was done with PCR products, which contained the gene of interest including 1 kb right and left borders.

For N-terminal tagging of hydrophobins or expression under *rodA* promoter control, hydrophobin open reading frames were cloned into pMCB17apx or pDM08 (pAGR06-11, and pTT07). In order to study the localization of the hydrophobins expressed from their native promoters, the *rodA* promoter in pTT07 was replaced by the respective hydrophobin promoters (pAGR14-18).

In order to follow the expression pattern during different developmental stages, spores from strain TN02A3 were cultivated over night in liquid minimal medium supplemented with uracil, uridine and pyro at 37°C. Afterwards the mycelium was collected and divided into five equal parts of which each was further cultivated on a cellophane layer on top of solid minimal medium with uracil, uridine and pyridoxin (pyro) at 37°C. All experiments were done in triplicate. Mycelium was collected after 6 h, 8 h, 12 h and 24 h respectively (**Fig. 3**). For the determination of the expression of the different hydrophobins under the control of the *rodA* promoter in a *ΔrodA* strain, spores of each strain (SAGR14, SAGR06, SAGR07, SAGR08, SAGR09, SAGR16) were spread on the surface of liquid minimal medium and incubated at 37°C for 24 h.

For RNA isolation mycelium was collected, shock-frozen in liquid nitrogen and lyophilized. RNA was extracted with the Fungal RNA Kit from Omega Bio-Tek following the manufacturers protocol. For DNA digestion the Ambion Turbo DNA Free Kit was used. For Realtime PCR the Bioline SensiFast SYBR and Fluorescein One Step Kit was used according to the manufacturers protocol. Two technical replicates were performed.

### Determination of the contact angle of droplets on *A. nidulans* colonies

The static water contact angles of colony surfaces were measured with an OCA20 by datapysics and the software SCA 202 v3.12.11. 10 μl drops (0.2% SDS, 50 mM EDTA) were put on the surface of the colonies and imaged with a CCD camera with a resolution of 768×576 px. The form of the drop was approached with an ellipse fit, allowing the determination of the water contact angle. The SDS solution was used to lower the surface-tension of the liquid, which makes it easier to discriminate between differences in wettability of the spores.

### Atomic force microscopy of the spore surface

Polished Silicon wafers (Siegert Consulting, monocrystalline, diameter: 150 mm, thickness: 675±25 μm, <100> orientation) were cut with a diamond cutter to samples with a size of about 1×1 cm^2^. These samples were gently pressed on the fungus that was taken as obtained. By this some fungus spores remained on the substrate surface and were investigated by AFM.

The used AFM was a MultiModeTM Nanoscope III (Veeco, Digital Instruments). AFM pictures were taken with cantilevers of Olympus, with a resonance frequency between 233 and 375 kHz and an average spring constant of 42 N/m. The scan sizes were 500×500 nm^2^, 750×750 nm^2^ and 1000×1000 nm^2^. As feedback control parameters we used an integral gain between 0.2 and 0.3, a proportional gain of 2.0 and an amplitude setpoint between 1.5 and 1.75 V. With the software Nanoscope 8.0 the AFM pictures were processed and the height, the length, the width and the bundling of the rodlets were investigated, each with 11 measured values per analyzed picture.

## Supporting Information

Table S1 ***A. nidulans*** **strains used in this study.** All strains contain the *veA1* mutation.
(DOCX)

Table S2 **Plasmids used in this study.**
(DOCX)

Table S3 **Oligonucleotides used in this study.** Restriction sites are underlined.
(DOCX)
