# Supplementary material for: Six Hydrophobins Are Involved in Hydrophobin Rodlet Formation in Aspergillus nidulans and Contribute to Hydrophobicity of the Spore Surface
Source: PLoS One. 2014 Apr 10;9(4):e94546. doi: 10.1371/journal.pone.0094546 (PMC3983194; doi:10.1371/journal.pone.0094546)
Supplement: Table S1 — A. nidulans strains used in this study. All strains contain the veA1 mutation. (DOCX) [file pone.0094546.s001.docx]

**Table S1: *A. nidulans* strains used in this study. All strains contain the *veA1* mutation.**

| **Name** | **Content** | **Source** |
| --- | --- | --- |
| GR5 | *pyrG89; wA3; pyroA4; veA1* | G. May, Houston, TX |
| RMS011 | *pabaA1, yA2; ∆argB::trpC∆B; trpC801, veA1* | [[1](#_ENREF_1)] |
| TN02A3 | *pyrG89; argB2; nkuA::argB; pyroA4; veA1* | [[2](#_ENREF_2)] |
| RMS019 | *rodA* deletion strain (RMS011 background) | [[1](#_ENREF_1)] |
| TMS027 | *dewA* deletion strain (RMS011 background) | [[3](#_ENREF_3)] |
| STT01 | *dewB* deletion strain (TN02A3 background)  PCR fusion construct, *pyrG* as marker | this study |
| STT02 | *dewC* deletion strain (TN02A3 background)  triple ligation of PCR fragments with *Sfi*I, *pyrG* as marker | This study |
| SAGR01 | *dewD* deletion strain (TN02A3 background)  knock-out cassette with *pyrG* as marker | FGSC, Kansas |
| SAGR12 | *dewE* deletion strain (TN02A3 background)  knock-out cassette with *pyrG* as marker | FGSC, Kansas |
| SAGR02 | Δ*rodA* (RMS019) re-complemented with *rodA*  ORF with 1 kb up- and downstream, co-transformed with pCK17 (*pabaA*) | This study |
| SAGR03 | Δ*dewA* (TMS027) re-complemented with *dewA*  ORF with 1 kb up- and downstream, co-transformed with pCK17 (*pabaA*) | This study |
| SAGR04 | Δ*dewB* (STT01) re-complemented with *dewB*  ORF with 1 kb up- and downstream, co-transformed with pNZ11 (*pyroA*) | This study |
| SAGR05 | Δ*dewC* (STT01) re-complemented with *dewC*  ORF with 1 kb up- and downstream, co-transformed with pNZ11 (*pyroA*) | This study |
| SAGR11 | Δ*dewD* (SAGR01) re-complemented with *dewD*  ORF with 1 kb up- and downstream, co-transformed with pNZ11 (*pyroA*) | This study |
| SAGR13 | Δ*dewE* (SAGR12) re-complemented with *dewE*  ORF with 1 kb up- and downstream, co-transformed with pNZ11 (*pyroA*) | This study |
| SAGR06 | Δ*rodA* (RMS019) transformed with *rodA(p)::dewA* (pAGR10), co-transformed with pCK17 | This study |
| SAGR07 | Δ*rodA* (RMS019) transformed with *rodA(p)::dewB* (pAGR06), co-transformed with pCK17 | This study |
| SAGR14 | Δ*rodA* (RMS019) transformed with *rodA(p)::rodA* (pAGR09), co-transformed with pCK17 | This study |
| SCOS170 | TN02A3 transformed with *rodA(p)::mRFP* (pAGR13) | This study |
| SCOS172 | GR5 transformed with *dewA(p)::mRFP::dewA* (pAGR14) | This study |
| SCOS173 | GR5 transformed with *dewB(p)::mRFP::dewB* (pAGR15) | This study |
| SCOS174 | GR5 transformed with *dewC(p)::mRFP::dewC* (pAGR16) | This study |
| SAGR19a | Δ*rodA* (RMS019) transformed with *dewD(p)::mRFP::dewD* (pAGR17a), co-transformed with pCK17 | This study |
| SCOS175 | GR5 transformed with *dewE(p)::mRFP::dewE* (pAGR18) | This study |
| SCOS171 | TN02A3 transformed with *alcA(p)::mRFP* (pDM08) | This study |
| STT08 | TN02A3 transformed with *rodA(p)::mRFP::rodA* (pTT07) | This study |

1. Stringer MA, Dean RA, Sewall TC, Timberlake WE (1991) *Rodletless*, a new *Aspergillus* developmental mutant induced by directed gene inactivation. Genes Dev 5: 1161-1171.

2. Nayak T, Szewczyk E, Oakley CE, Osmani A, Ukil L, et al. (2006) A versatile and efficient gene targeting system for *Aspergillus nidulans*. Genetics 172: 1557-1566.

3. Stringer MA, Timberlake WE (1994) *dewA* encodes a fungal hydrophobin component of the *Aspergillus* spore wall. Mol Microbiol 16: 33-44.
