# Supplementary material for: Six Hydrophobins Are Involved in Hydrophobin Rodlet Formation in Aspergillus nidulans and Contribute to Hydrophobicity of the Spore Surface
Source: PLoS One. 2014 Apr 10;9(4):e94546. doi: 10.1371/journal.pone.0094546 (PMC3983194; doi:10.1371/journal.pone.0094546)
Supplement: Table S2 — Plasmids used in this study. (DOCX) [file pone.0094546.s002.docx]

**Table S2: Plasmids used in this study.**

| **Name** | **Content** | **Source** |
| --- | --- | --- |
| pJET1.2 | PCR cloning vector | Fermentas |
| TOPO pCR2.1 | PCR cloning vector | Invitrogen |
| pNZ11 | *pyroA* ORF from *A. nidulans* in TOPO pCR2.1 | This study |
| pCK17 | *pabaA* ORF plus 1 kb up- and downstream in pJET1.2 | This study |
| pCMB17apx | *alcA(p)::GFP*, for N-terminal fusion of GFP; contains *N. crassa pyr4* | [[1](#_ENREF_1)] |
| pDM08 | *alcA(p)::mRFP*, for N-terminal fusion of mRFP; contains *N. crassa pyr4* | [[2](#_ENREF_2)] |
| pAGR06 | *rodA(p)::dewB* in pCMB17apx, oligonucleotides no. 15 and 3 | This study |
| pAGR09 | *rodA(p)::rodA* in pCMB17apx, oligonucleotides no. 15 and 1 | This study |
| pAGR10 | *rodA(p)::dewA* in pCMB17apx, oligonucleotides no. 15 and 2 | This study |
| pAGR13 | *rodA(p)::mRFP* in pDM08, *rodA* deleted and blunt end ligated (pTT07 cut with AscI and PacI, blunt ended and religated) | This study |
| pAGR14 | *dewA(p)::mRFP::dewA* in pDM08, oligonucleotides 10 + 5 | This study |
| pAGR15 | *dewB(p)::mRFP::dewB* in pDM08, oligonucleotides 11 + 6 | This study |
| pAGR16 | *dewC(p)::mRFP::dewC* in pDM08, oligonucleotides 12 + 7 | This study |
| pAGR17 | *dewD(p)::mRFP::dewD* in pDM08, oligonucleotides 13 + 8 | This study |
| pAGR18 | *dewE(p)::mRFP::dewE* in pDM08, oligonucleotides 14 + 9 | This study |
| pTT07 | *rodA(p)::mRFP::rodA* in pDM08, oligonucleotides 15 + 4 | This study |
|  |  |  |

1. Efimov V, Zhang J, Xiang X (2006) CLIP-170 homologue and NUDE play overlapping roles in NUDF localization in *Aspergillus nidulans*. Mol Biol Cell 17: 2021-2034.

2. Veith D, Scherr N, Efimov VP, Fischer R (2005) Role of the spindle-pole body protein ApsB and the cortex protein ApsA in microtubule organization and nuclear migration in *Aspergillus nidulans*. J Cell Sci 118: 3705-3716.
