# Supplementary material for: Six Hydrophobins Are Involved in Hydrophobin Rodlet Formation in Aspergillus nidulans and Contribute to Hydrophobicity of the Spore Surface
Source: PLoS One. 2014 Apr 10;9(4):e94546. doi: 10.1371/journal.pone.0094546 (PMC3983194; doi:10.1371/journal.pone.0094546)
Supplement: Table S3 — Oligonucleotides used in this study. Restriction sites are underlined. (DOCX) [file pone.0094546.s003.docx]

**Table S3: Oligonucleotides used in this study. Restriction sites are underlined.**

| **no** | **Name** | **Sequence** | **Plasmid** |
| --- | --- | --- | --- |
| **1** | RodA_oSP_KpnI_ forward  RodA_PacI_reverse | CAGGTACCCCTCCTGCCCATGATTCCC  GTTAATTAATTAGAGGATGGAGCCAAGGGCAACGC | pAGR09 |
| **2** | DewA_oSP_KpnI_ forward  DewA_PacI_reverse | GAGGTACCCTCCCGGCCTCTGCCG  CTTAATTAATTACTCAGCCTTGGTACCGG | pAGR10 |
| **3** | DewB_oSP_KpnI_ forward  DewB_PacI_reverse | CTGGTACCGACAAGTTCCCCGTCCCC  CTTAATTAATTACAGAATGGAGCCAAGGGC | pAGR06 |
| **4** | RodA_AscI_forward  RodA_PacI_reverse | TAGGCGCGCC CCTCCTGCCCAT  GTTAATTAATTAGAGGATGGAGCCAAGGGCAACGC | pTT07 |
| **5** | DewA_AscI_forward  DewA_PacI_reverse | AGGCGCGCCTCTCCCGGCCTCTGCCG  CTTAATTAATTACTCAGCCTTGGTACCGG | pAGR14 |
| **6** | DewB_AscI_forward  DewB_PacI_reverse | AGGCGCGCCTGACAAGTTCCCCGTCCCC  CTTAATTAATTAGCCGCCACTCTTCGCAC | pAGR15 |
| **7** | DewC_AscI_forward  DewC_PacI_reverse | AGGCGCGCCTGCCCCCCATGCCCCCGG  GTTAATTAATTAGAGAACCTGGACAGGAACAC | pAGR16 |
| **8** | DewD_AscI_forward  DewD_PacI_reverse | TAGGCGCGCCTCGACCGTACTTCTGCCCTCTAG  GCATTAATTAACTACTTGTCAACGCCATCAC | pAGR17 |
| **9** | DewE_AscI_forward  DewE_PacI_reverse | TAGGCGCGCCTAACTCTGAGAAGCGGCAAAGC  GCATTAATTAATTAGTGGCCGTGCTCCAG | pAGR18 |
| **10** | pDewA_EcoRI_forward  pDewA_KpnI_reverse | GTCAGAATTCGCCTATGCTTCTCGTCGCAAAAGTGGAT  CAGGTACCGCGGTTGCGGTGGCCGCGG | pAGR14 |
| **11** | pDewB_EcoRI_forward  pDewB_KpnI_reverse | GTCAGAATTCGGTCGTGGCTCAGGTTCGCTGAGA  CAGGTACCGCGCTGGCGAGCGCAAAGA | pAGR15 |
| **12** | pDewC_XhoI_forward  pDewC_KpnI_reverse | GTCACTCGAGGCAGCCGATATCACATTCAGAGCCT  CAGGTACCTGCGCTGGCCATTTGCAGTCCAA | pAGR16 |
| **13** | pDewD_EcoRI_forward  pDewD_KpnI_reverse | GTCAGAATTCGCTCCTCGACTACGTTTACACTGCC  CAGGTACCTCCGGCCATTGTTGGCCCGGC | pAGR17 |
| **14** | pDewE_EcoRI_forward  pDewE_KpnI_reverse | GTCAGAATTCCGTCTCTGGAGCAGTCTTACTGATAAGG  CAGGTACCGGCAGCGGAGGGCAAAGCAG | pAGR18 |
| **15** | BsaI_EcoRI_(nP)rodA_fwd  (nP)rodA(SP)_KpnI_rev | GGTCTCGAATTCCAAGTTTGAGGTTATTTTCTGG  CAGGTACCGAGGGCCGCGACGGAGGCGGCG | pAGR06, pAGR09, pAGR10, pTT07 |
|  | **For gene deletions:** |  |  |
| **16** | Ko_dewC_P1_fwd  Ko_dewC_P3_rev | ATGACGCCAAAGTCTTACTACAC  TGGCCGCGTTGGCCTTGATGATGATGATGACAAGG |  |
| **17** | Ko_dewC_P4_fwd  Ko_dewC_P6_rev | TGGCCTGAGTGGCCCGGAATTTATCTCACGCTTTG  CCCAGTACAGGAACAGAGTC |  |
| **18** | Ko_dewC_P2_fwd  Ko_dewC_P5_rev | AGGATACCATCTTGCGTATG  AGCTTAACAGCAATGGTAGG |  |
|  | **For re-complementation:** |  |  |
| **19** | cRodA fwd  cRodA rev | CAGAGGTCAGTATCAGCCAC  CGCATGGGCATTGAATGAGAG |  |
| **20** | cDewA fwd  cDewA rev | GCGATGCTACTGGTTGTTCG  AGCGGTCAACTGCCAGTCAT |  |
| **21** | cDewB fwd  cDewB rev | GAAGCCAGATGCCCGCAAT  CATCTATAGACTGCGCGCTTGAAC |  |
| **22** | cDewC fwd  cDewC rev | GAAGCGCATGTCAGTGACTG  CCTTAACGATCGGCAAGCCA |  |
| **23** | cDewD fwd  cDewD rev | CTACGATTGGTACACCGGCGCCGT  ACCGCTTTAACTCCAGCAATTGCACCC |  |
| **24** | cDewE fwd  cDewE rev | CTCGGTGACTAGCTCGTATGATCA  CGGGAGAGGACGGTTTGTCTGTTC |  |
|  | **For expression analyses:** |  |  |
| **25** | RodA-RT fwd  RodA-RT rev | GTTCGCTGGCAATGGTGTTGGC  CCAGACAGAAGACCCTCATCAACG |  |
| **26** | DewA-RT fwd  DewA-RT rev | GAAGGCACCACCTGCAATGTCG  CTGGTCAATCAAGCTCGCCTTGG |  |
| **27** | DewB-RT fwd  DewB-RT rev | GTGCTTTCAGCGGTTGCTCGAAG  CAGAATGGAGCCAAGGGCAATGC |  |
| **28** | DewC-RT fwd  DewC-RT rev | CGCTTCCGTTGAGGGTATTCTCG  CGAGGCCGTTCTGTATACCATCAG |  |
| **29** | DewD-RT fwd  DewD-RT rev | CATCTTTCCACCTCCGCTGCTG  GACTCCCTCAAACGAGACCTTGG |  |
| **30** | DewE-RT fwd  DewE-RT rev | GTTCTGCTCTGGCTTCTGCTTTGC  CGATTGCAGCTGCGTCGATACC |  |
| **31** | H2b fwd  H2b rev | CTGCCGAGAAGAAG CCTAGCAC  GAAGAGTAGGTCTCCTTCCTGGTC |  |
